# Genetic estimators of DNA methylation provide insights into the molecular basis of polygenic traits

**DOI:** 10.1038/s41398-017-0070-x

**Published:** 2018-01-31

**Authors:** Virginie Freytag, Vanja Vukojevic, Holger Wagner-Thelen, Annette Milnik, Christian Vogler, Markus Leber, Leonie Weinhold, Anne C. Böhmer, Steffi Riedel-Heller, Wolfgang Maier, Dominique J.-F. de Quervain, Alfredo Ramirez, Andreas Papassotiropoulos

**Affiliations:** 1 Division of Molecular Neuroscience, Department of Psychology, University of Basel, CH-4055 Basel, Switzerland; 2 Transfaculty Research Platform Molecular and Cognitive Neurosciences, University of Basel, CH-4055 Basel, Switzerland; 3 Department Biozentrum, Life Sciences Training Facility, University of Basel, CH-4056 Basel, Switzerland; 4 Department of Psychiatry and Psychotherapy, University of Bonn, Bonn, Germany; 5 Department for Neurodegenerative Diseases and Geriatric Psychiatry, University Hospital Bonn, Bonn, Germany; 6 Psychiatric University Clinics, University of Basel, CH-4055 Basel, Switzerland; 7 Department of Psychiatry and Psychotherapy, University of Cologne, Cologne, Germany; 8 Department of Medical Biometry, Informatics and Epidemiology, University Hospital Bonn, Bonn, Germany; 9 Institute of Human Genetics, University of Bonn, Bonn, Germany; 10 Department of Genomics, Life and Brain Center, University of Bonn, Bonn, Germany; 11 Institute of Social Medicine, Occupational Health and Public Health, University of Leipzig, Leipzig, Germany; 12 German Center for Neurodegenerative Diseases (DZNE), Bonn, Germany; 13 Division of Cognitive Neuroscience, Department of Psychology, University of Basel, CH-4055 Basel, Switzerland

## Abstract

The large biological distance between genetic risk loci and their mechanistic consequences in the tissue of interest limits the ability to establish functionality of susceptibility variants for genetically complex traits. Such a biological gap may be reduced through the systematic study of molecular mediators of genomic action, such as epigenetic modification. Here, we report the identification of robust genetic estimators of whole-blood CpG methylation, which can serve as intermediate molecular traits amenable to association testing with other genetically complex traits. We describe the relationship between these estimators and gene expression, demonstrate their genome-wide applicability to association testing even in the absence of individual genotypic data, and show that these estimators powerfully identify methylation-related genomic loci associated with polygenic traits and common diseases, such as schizophrenia. The use of genetic estimators for blood DNA methylation, which are made publically available, can serve as a valuable tool for the identification of epigenetic underpinnings of complex traits.

## Introduction

Improving understanding, diagnosis, and therapy of human disease has been one of the central promises of the human genome project^1^. This promise is being increasingly fulfilled. For example, cancer research has benefited dramatically from the discoveries related to the human genome^2^, mainly because the genomic mechanisms leading to the development of many cancers are amenable to direct observation. However, the situation is slightly different in disorders for which the underlying molecular events are not easily accessible, as is the case for mental disorders^3^. Advances in the development of high-throughput genotyping and analytical software, and the launch of large collaborative efforts have led to the identification of numerous well-validated genetic risk factors for such common disorders. However, the functional relevance of most discovered loci and the molecular mechanisms behind the reported genetic association signals remain elusive^4^.

One of the main reasons for the limited ability to establish functionality of susceptibility variants is the large biological distance between a genetic polymorphism and its related mechanistic consequences in the tissue of interest. Such biological gap may be reduced by the study of molecular mediators of genomic action, such as gene expression^4^. For example, in such common neuropsychiatric disorders as schizophrenia, genetic susceptibility variants are significantly enriched in promoter and enhancer regions and point to a functional link between disease-associated noncoding single-nucleotide polymorphisms (SNPs) and transcriptional regulation in the brain^5^. The integration of transcriptomics data in the study of the genetic factors of complex traits has significantly improved our understanding of their genetic basis^6^. Thus, methods that reduce the gap between genetic susceptibility and its functional consequences are expected to increase our understanding of the genetic underpinnings of genetically complex traits.

Genetic estimators for gene expression have been recently proposed in this context^4,7,^. These methods capitalize on the joint additive effects of *cis*-markers on a given expression trait to estimate gene expression from individual genotypes. At the population level, the derived genetic estimates represent an intermediate molecular trait, amenable to association testing with the phenotype under study. This approach can be viewed as genetic correlation testing for which a significant association is interpreted as existence of shared co-localizing genetic factors between the complex phenotype and the investigated expression trait.

Here we report the generation of robust genetic estimators of epigenetic regulation as an attempt to provide insights into the molecular basis of polygenic traits by minimizing the biological gap between genetic variation and its functional impact. We focused on DNA methylation (specifically on the methylation of 5′-C-phosphate-G-3′ (CpG) sites), the most extensively studied epigenetic modification to date, which directly regulates important molecular processes such as gene expression, imprinting, and chromosomal inactivation^8–10^. High-throughput methylomic profiling studies have highlighted the strong local genetic regulation of DNA methylation^11–15^, with possibly multiple co-localized markers contributing independently to variation in DNA methylation at individual CpG sites^16^.

We generated genetic estimators of DNA methylation (DNAm), that allow testing for localized shared genetic contributions between DNAm variation and complex traits. We demonstrate their applicability even to studies providing summary SNP statistics only, and show exemplarily that such estimators result in the identification of epigenetic underpinnings of a common neuropsychiatric disease.

## Materials and methods

### Study datasets

Whole-blood methylomic profiles and genotypic data were obtained from healthy young adults recruited in the course of two separate studies conducted in Basel, previously described^17^, and from elderly adults (ACD BONN sample, see below).

The Basel Imaging dataset (BASEL1) included *N* = 533 participants (age range: 18–37 years old; 222 males) and the independent Basel Cognitive dataset (BASEL2) included a total of *N* = 319 participants (age range: 18–37 years old; 97 males). The study protocols were approved by the ethics committee of the cantons of Basel-Stadt and Basel-Landschaft. All participants gave written informed consent after complete description of the study protocols. Subjects were free of any neurological or psychiatric condition and did not take medication at the time of the experiment.

The ongoing German Study on Ageing, Cognition, and Dementia (ACD) started in 2003 and consists of 3327 non-demented elderly subjects over 75 years of age who were randomly selected from the general-practice registry in six German cities (Bonn, Dusseldorf, Hamburg, Leipzig, Mannheim, and Munich)^18^. The entire study protocol was approved by the local ethics committees at the University of Bonn, Bonn, Germany; the University of Hamburg, Hamburg, Germany; the University of Duesseldorf, Duesseldorf, Germany; the University of Heidelberg/Mannheim, Mannheim, Germany; the University of Leipzig, Leipzig, Germany; and the Technical University of Munich, Munich, Germany. Before participation written informed consents were collected from all subjects. Participants were followed-up in 1.5-year intervals and received cognitive testing at each follow-up^19^. Out of the ACD cohort a subsample comprising 302 subjects (age range: 79–94 years old; 88 males) with available whole-blood methylomic data and genotypic data was used in this study. After methylomic and genotypic samples quality control, a total of *N* = 288 subjects (mean age: 84.2, s.d. 2.9; 82 males) were included in analysis. Subjects were free of any neurological or psychiatric condition.

### Methylomic profiling

#### BASEL1 and BASEL2 datasets

A detailed description of methylomic profiling protocols can be found in Milnik et al.^17^. Briefly, methylomic profiling was performed using the Illumina HumanMethylation450 array. Samples of non-European ancestry were identified using Hapmap references population genotypes and excluded from analysis (*n* = 35 in BASEL1 sample, yielding *N* = 533 remaining for analysis; none identified in BASEL2 sample). The β-values were calculated from SWAN normalized intensities^20^. Subsequently, β-values were *M*-transformed and adjusted for processing plate effect (*z*-transformation), age, sex, and the main sources of technical variations inferred from principal components analysis^17^. The β-values with detection *p*-value > 0.05 were considered as missing. Individual CpG sites were excluded based on the following criteria: non-CpG context, non-autosomal probes, probes with a SNP mapping to the target CpG site or with three or more SNPs within the 50-mer probe (minor allele frequency (MAF )> 0.01) (based on RnBeads package annotation), multi-mapping or polymorphic CpGs (MAF > 0.01 in European population) reported in refs.^21,22^, and probes with missing rate ≥5% in final samples. Prior to analysis, missing values were imputed using the R package impute (https://bioconductor.org/packages/release/bioc/html/impute.html) with *k* = 10.

#### ACD BONN dataset

The Illumina HumanMethylation450 array was used to quantify DNA methylation in bisulfite converted genomic DNA from whole-blood samples. Each sample consisted of one technical probe. Subjects were excluded based on the following criteria: (1) inconsistency between reported and methylome estimated gender (two subjects excluded); (2) exceeding more than four s.d. from the mean on one of the two first principal components (one subject excluded); (3) inconsistency between actual genotypes and those inferred from methylomic data (none excluded); and (4) subjects with more than 1% of sites with detection *p* > 0.05 (none detected). An additional subject processed on a single plate was further excluded from analysis. This yielded a total *N* = 297 samples for analysis. Methylation values were pre-processed using the wateRmelon package^23^. First, CpG sites with beadcount <3 in 5% of samples and/or sites with detection *p* > 0.05 in 1% of study samples were excluded. The β-values were next normalized using the dasen algorithm, logit transformed, adjusted for technical covariates (plate and Sentrix ID) using ComBat^24^, and finally adjusted for age, sex, and the six first principal component analysis axes.

#### Cell composition adjustment

Cell type proportion estimates (CD4 T cells, CD8 T cells, natural killers, granulocytes, monocytes, and lymphocytes B) were obtained for each sample using the EpiDISH-CIBERSORT approach^25^. In each testing dataset, we compared EstiMeth model performance before and after further linear regression adjustment of DNAm for the estimated cell type proportions.

### Genotyping

#### BASEL1 and BASEL2 datasets

DNA was isolated from saliva sample and genotyped using the Affymetrix Genome-Wide Human SNP array 6.0 following the manufacturer’s protocol. Genotype imputation was performed independently for each BASEL1 and BASEL2 dataset, on the University of Michigan Imputation Server^26^ (settings for marker imputation: MAF > 0.01, call rate > 95%). In the BASEL1 dataset, approximately 5 million imputed SNPs with MAF > 0.05, Hardy–Weinberg equilibrium (HWE) *p*-value > 0.0001, and imputation score *R*^2^ > 0.8 were retained for training genetic models of DNAm estimation. To allow complete evaluation of the trained models, all selected markers were considered in the BASEL2 dataset.

#### ACD BONN dataset

DNA was isolated from whole-blood samples and genotyped using the Illumina Infinium OmniExpressExome-8v1.3 Kit following the manufacturer’s protocol. Illumina’s GenomeStudio software was used to export the genotyping raw data into plink format. Variant and sample quality control was performed before imputation. Samples were excluded if their ancestry was non-European, if samples were related, if they had an outlying heterozygosity rate, or if they had a sample call rate < 98%. SNPs with a HWE *p*-value > 10^−6^, a MAF > 0.01, and a call rate > 95% were included. This led to a SNP reduction from 932,703 to 606,260 before the imputation. Genotype imputation was performed on the University of Michigan Imputation Server^26^.

For evaluation of EstiMeth models, SNPs were filtered out based on the following criteria: (1) inconsistency of allele pairs between EstiMeth models and imputed genotypes, (2) MAF < 0.01, and (3) genotype imputation *R*^2^ < 0.5. Models with less than 50% SNP coverage were discarded from analysis (*n* = 1061), yielding a total of 85,170 models evaluated.

### EstiMeth model implementation

A total of 395,014 CpGs, measured in both BASEL1 and BASEL2 datasets and encompassed by more than one *cis*-SNPs within ±1 Mbp, were considered for analysis. At each of these individual CpG sites, an elastic net^27^ genetic additive model was fitted between all encompassing imputed *cis*-SNPs, and adjusted DNA methylation signal.

Genotypes were coded as: 0: homozygous for the major allele, 1: heterozygous, and 2: homozygous for the minor allele. Models were implemented using the glmnet R package^28^ with α elastic net constraint fixed to 0.5. Default standardization of genotypes (mean centering and unit variance) was applied within the training procedure which resulted in slight improvement of model performance (Supplementary Table 1). The β-coefficients were returned on the original genotype scale. The λ tuning parameter was determined using a 10-fold cross-validation scheme. This modeling allowed simultaneous shrinkage of individual β-coefficients and selection of variables, thus drastically reducing the number of SNPs finally included in each model (average *n* = 3522 SNPs before selection, average *n* = 26 SNPs after selection across all non-null models).

Model performance was assessed using Pearson’s squared correlation *r*^2^ between the model estimate—linear combination between elastic net inferred β-coefficients and observed genotypes—and the actual adjusted DNAm signal; for the training dataset, *r*^2^ refers to cross-validation performance.

We derived a set of robust genotype-based estimators for DNAm—i.e. EstiMeth models—as follows: (1) all models exhibiting a significant association between the elastic net-derived cross-validation estimator and the actual values in the BASEL1 training dataset (false discovery rate (FDR) < 0.05 across all non-null models); (2) among those, all models exhibiting a significant association between the elastic net-derived estimator and the actual values in the BASEL2 testing dataset (FDR < 0.05); (3) all models resulting in a positive correlation between actual and genotype-based estimated value in the testing dataset. This yielded a total of 86,710 models likely reflecting a robust genetically driven DNAm signal at the corresponding CpG (minimum observed *r*^2^ in training dataset = 0.94%; minimum *r*^2^ in testing dataset = 1.37%).

### MetaMeth implementation

#### Statistical model

We relied on the approach recently proposed by Barbeira et al.^29^ for estimating genetic correlation between EstiMeth model and a trait based on genome-wide association study (GWAS) summary statistics solely. Specifically, consider a given EstiMeth model comprising weights *W* at *p* SNPs. Let *T*_*g*_ denote the *t*-value between the EstiMeth linear combination and the trait. Let ∑_*p*_ be the observed covariance matrix of the *p* SNPs, and *Z* the vector of standardized coefficients obtained from testing each SNP for association with the trait (GWAS summary statistics, *β/se(β)*).

As described by Barbeira et al.^29^, *T*_*g*_ is equivalent to *Z*_*g*_:

1 $$T_g = Z_g = \mathop {\sum}\limits_{k = 1}^p {w_k} \frac{{\hat \sigma _k}}{{\widehat {\sigma _g}}}\frac{{\widehat {\beta _k}}}{{se(\widehat {\beta _k})}}\sqrt {\frac{{1 - R_k^2}}{{1 - R_g^2}}}$$

with

$$\widehat {\sigma _g} = \sqrt {W\prime {\mathrm{\Sigma }}_pW}$$

$\hat \sigma _k$ the standard deviation at SNP *k*;

$R_k^2$ the proportion of phenotypic variance explained by SNP *k*;

$R_g^2$ the proportion of phenotypic variance explained by EstiMeth estimator.

In the absence of genotypes, the $R_g^2$ term cannot be estimated and the covariance structure ∑_*p*_ has to be estimated from a reference population, which leads to the approximation:

2 $$T_g \approx MetaMeth\,Z_g = \mathop {\sum}\limits_{k = 1}^p {w_k} \frac{{\hat \sigma _k}}{{\widehat {\sigma _{gref}}}}\frac{{\widehat {\beta _k}}}{{se(\widehat {\beta _k})}}$$

with

$$\widehat {\sigma _{gref}} = \sqrt {W\prime {\mathrm{\Sigma }}_{pref}W}$$

This approximation has two potential caveats.

Firstly, as pointed by Barbeira et al.^29^, removal of the *R*^*2*^ ratio can lead to remarkable underestimation of *T*_*g*_ for SNPs with large effect sizes. This deviation was notably observed when comparing EstiMeth and MetaMeth approaches on DNAm signal, which implicate large effect sizes. Considering the actual sample’s covariance matrix, the exact statistic derived from Eq. 1 is equal to *T*_*g*_ (Supplementary Figure 7-A). Removal of the *R*^*2*^ ratio in Eq. 2, while still using the exact sample’s covariance matrix, leads to deviation from the original *T*_*g*_ with a global decrease of derived statistics (Supplementary Figure 7-B). The same observation was drawn from the power study presented in Supplementary Figure 6.

Secondly, the divergence between the reference population and actual covariance structures can lead to biased estimates. Using Eq. 2, we observed inflation of genome-wide level Type I error (Supplementary Figure 5). To account for this uncertainty, we penalized the denominator $\widehat {\sigma _g}$ by multiplying the diagonal of the ∑_*pref*_ matrix (${\mathrm{\Sigma }}_{pref} = {\mathrm{\Sigma }}_{pref} + {\mathrm{\lambda }}_s{\mathrm{diag}}\left( {{\mathrm{\Sigma }}_{pref}} \right)$ with *λ*_*s*_ = 0.1)^7^. We found empirically* λ*_*s*_ = 0.1 to achieve conservative results, at the cost of decreased power. Unless otherwise specified, all reported results were obtained using *λ*_*s*_ = 0.1

#### 1000G reference panel

The SNPs covariance matrices *∑*_*pref*_ were inferred from the publicly available 1000G reference genotypes (http://ftp.1000genomes.ebi.ac.uk/vol1/ftp/release/20130502/) considering *N* = 503 samples from European populations. SNPs with allele pair mismatching alleles observed in the BASEL1 dataset were discarded, yielding a total of 5,059,361 markers referred as 1000G SNP panel (99% of markers from BASEL1 dataset). Elastic net models were re-trained on the BASEL1 sample using the 1000G SNP panel. Models not reaching the minimum *r*^2^ initially observed in the training and BASEL2 testing datasets were excluded from MetaMeth benchmarking analyses (*n* = 86,518 models remaining out of 86,710). Overall, we observed comparable performance of the EstiMeth models implemented on the BASEL1 and 1000G SNP panels (correlation between cross-validation *r*^2^ across all EstiMeth CpGs > 0.99; correlation between BASEL2 testing *r*^2^ > 0.99).

#### Hapmap reference panel

To ensure sufficient coverage of EstiMeth models for the MetaMeth scan of GWAS summary statistics imputed from HapMap reference panel, we additionally provide EstiMeth models inferred from a restricted SNP panel. Elastic net models were re-trained on the BASEL1 sample using the SNPs overlapping between 1000G, HapMap SNPs, and BASEL1 imputed SNPs (*n* = 2,228,898 markers). Models reaching the minimum *r*^2^ initially observed in the training and BASEL2 testing datasets were retained, yielding *n* = 82,885 models, showing performance comparable to the EstiMeth models implemented on the BASEL1 dataset (correlation between cross-validation *r*^2^ across all EstiMeth CpGs > 0.99; correlation between BASEL2 testing *r*^2^ > 0.99).

#### Simulation studies

Type I error rate was assessed on 1000 repeats of genome-wide MetaMeth scan using phenotypes randomly generated from a normal distribution. The power study was performed by generating, for each CpG, a phenotype showing an average *r* = 0.27 with the EstiMeth estimate. This corresponds to an effect size of 7.1%, detectable with 50% power considering the BASEL2 sample size *N* = 319 and genome-wide significance threshold *α* = 0.05/86,518. This procedure was repeated 300 times per CpG.

### Transcriptomic analyses

#### Data processing

Blood samples were collected using PAXgene Blood RNA Tubes (PreAnalytix Qiagen/BD, Switzerland). Expression profiles were obtained for *N* = 408 individuals of the BASEL1 sample using the Affymetrix GeneChip Human Transcriptome Array 2.0 (see Supplementary text), providing quantification of expression levels for ~67K transcript clusters (referred as genes). Individual expression values were adjusted for age and sex using linear regression. Expression signals were adjusted for unknown technical confounders while preserving local genuine genetic effects. This was achieved by examining the number of identified *cis*-expression quantitative trait loci (eQTL) while further adjusting expression values for increasing number of principal components;^30,31^ this procedure was repeated until no increase in the number of identified eQTLs was observed anymore, leading to 23 components retained for final adjustment of expression values. Only genes annotated to RefSeq identifiers were considered for analysis. The annotation was based on the manufacturer’s information (GPL17586-45144) curated using the UCSC database version Oct 2015. This yielded a total of 21,186 autosomal genes entering subsequent analyses.

#### Association testing between DNAm and expression traits

The relationship between DNAm and expression traits was examined considering for each gene all CpGs located within ±1 Mbp from gene boundaries (*N* = 397,731 sites out of 397,947 CpGs from BASEL1 sample). Statistical association testing was performed using Pearson’s correlation test. Genome-wide significant associations were identified using Benjamini–Hochberg FDR correction. Expression signals were optimally processed for preserving genetic effects (see paragraph Data processing). In order to check whether this procedure possibly biased the over-representation of EstiMeth genetically driven CpGs among identified associations, the genome-wide CpG-gene association scan was re-conducted on expression data adjusted for main confounders only (batch effect adjustment using ComBat method implemented in the sva R package^24^ age, sex, and the seven first principal component axes that showed strong association with blood cell subtype composition). This analysis led to the identification of 11,760 significant associations (FDR < 0.05), implicating 8530 CpGs, among which 74% involved EstiMeth CpGs, convergent with results obtained from the primary analysis.

#### Genetic association analysis of DNA methylation and gene expression

For ensuring independence of the expression trait and the EstiMeth models, all models were re-trained on the BASEL2 dataset using the same methodology as for the initial EstiMeth implementation. Out of 86,710 models, a total of 83,337 non-null models could be inferred in the BASEL2 dataset and entered subsequent analyses. In turn, estimated DNAm values were obtained in the BASEL1 dataset using these EstiMeth models implemented on the BASEL2 dataset. These estimated values were subsequently tested for association with expression trait at their co-localizing gene(s). DNAm-expression associations were also examined under adjustment for EstiMeth models: DNAm was adjusted for EstiMeth estimated values using linear regression, and next tested for association with gene expression.

### MetaMeth application to GWAS summary statistics

GWAS summary statistics of Psychiatric Genomics Consortium (PGC) schizophrenia (SCZ) analysis (52 samples; 34,241 cases, 45,604 controls and 1235 parent-affected offspring trios)^32^ were downloaded from https://www.med.unc.edu/pgc/files/resultfiles/scz2.snp.results.txt.gz. GWAS summary statistics for inflammatory bowel disease (IBD) obtained from meta-analysis across 34,652 European samples^33^ were downloaded from https://www.ibdgenetics.org/downloads.html, file: EUR.IBD.gwas_info03_filtered.assoc); summary statistics for rheumatoid arthritis (RA) obtained from meta-analysis across 58,284 European samples^34^ were downloaded from http://plaza.umin.ac.jp/~yokada/datasource/software.htm (file: RA_GWASmeta_European_v2.gz); summary statistics for Alzheimer’s disease (AD) obtained from meta-analysis across 54,162 European samples^35^ were downloaded from http://web.pasteur-lille.fr/en/recherche/u744/igap/igap_download.php (file: IGAP_stage1.txt); summary statistics for Height obtained from meta-analysis across 253,288 European samples^36^ were downloaded from http://portals.broadinstitute.org/collaboration/giant/index.php/GIANT_consortium_data_files#GWAS_Anthropometric_2014_Height. MetaMeth scans for SCZ, AD, IBD, and RA were conducted considering EstiMeth models implemented on the BASEL1 SNP panel (*n* = 86,710) and corresponding SNP covariance structure inferred from BASEL1 dataset. MetaMeth analysis of height GWAS summary statistics was conducted considering EstiMeth models implemented on the HapMap SNP panel (*n* = 82,885) and covariance structure inferred from BASEL1 dataset. SNPs with allelic mismatch between GWAS report and EstiMeth models were discarded. For each analysis, models reaching a SNPs coverage greater or equal to 80% (e.g., percentage of SNPs included in EstiMeth overlapping with GWAS SNPs) were retained (SCZ: *n* = 86,380; AD: *n* = 84,288; Height: *n* = 72,532; IBD: *n* = 86,567; RA: *n* = 85,620). For each analysis, multiple testing adjustment was performed using Bonferroni method for the number of tests conducted.

#### Analysis of correlation patterns between whole blood and brain

We downloaded summary statistics of concordance of CpG signals between blood and brain from (https://redgar598.shinyapps.io/BECon/ (*n* = 413,466 CpGs)^37^. Briefly, these metrics report, for each CpG, the coefficient of variation (CV) of β-values in whole blood, and correlation coefficients between blood and each of three brain tissues (Brodmann areas 7, 10, and 20), derived from 63 paired methylomic samples (16 unique subjects). As reported in the original publication^37^, a CpG was considered as potentially informative between blood and brain tissues if it met the following criteria: (1) sufficient inter-individual variability in whole-blood DNAm characterized by a CV > 0.1; (2) concordance between whole blood and at least one brain tissue, characterized by absolute Spearman's correlation value >0.36 for BA7, >0.4 for BA10, and >0.33 for BA20 (*n* = 39,360). Significance of enrichment of informative CpGs across EstiMeth CpGs was assessed using 10,000 permutations, each of which consisted of counting the number of informative CpGs within a randomly sampled *n*-set of CpGs (*n* equals to number of EstiMeth CpGs).

### Code availability

The EstiMeth genetic models are made publicly available at http://mcn.unibas.ch/files/EstiMeth_Distribution.zip.

## Results

### Estimation of genetically driven DNA methylation

**Fig. 1:**
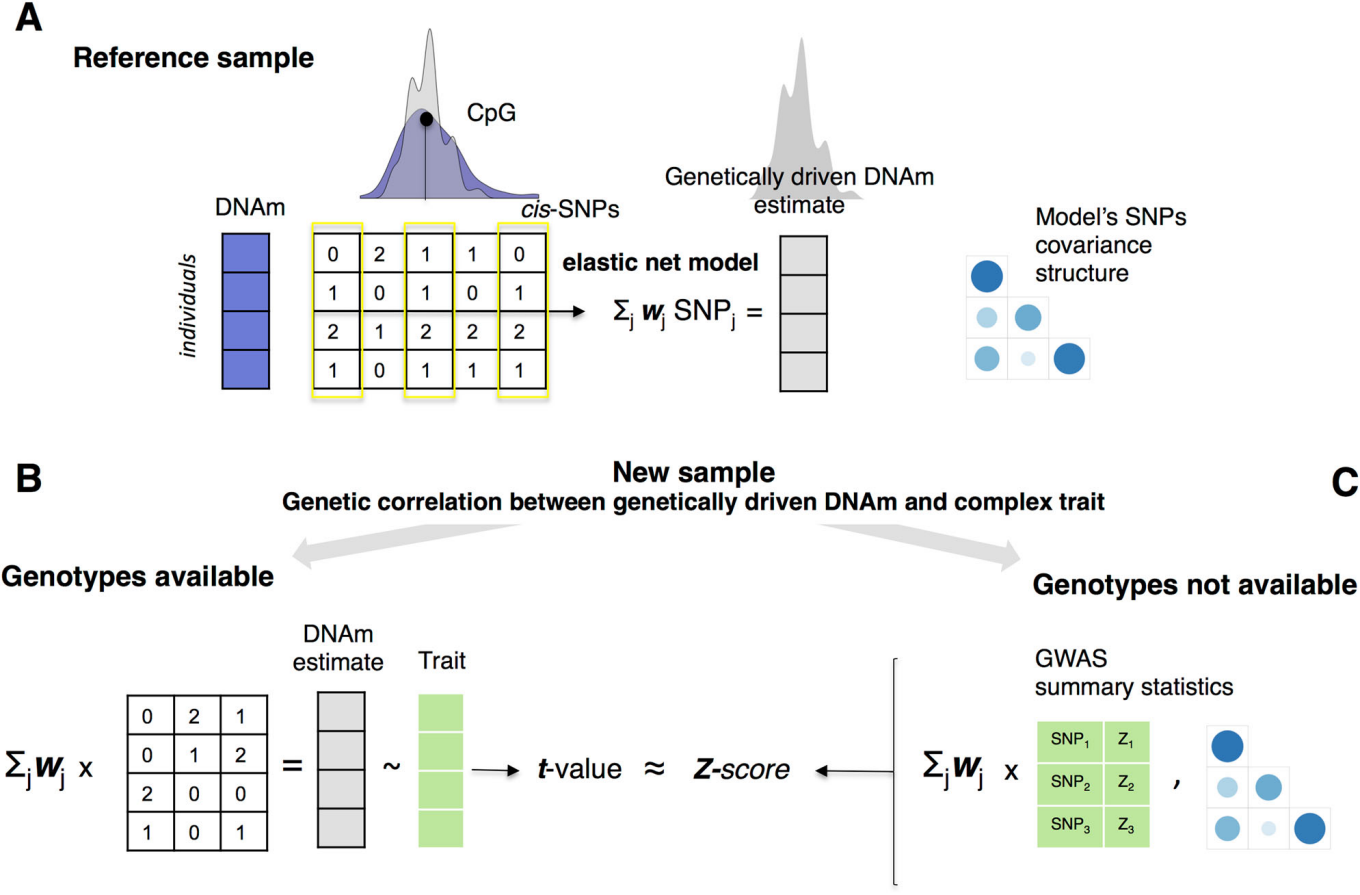
Estimation of genetically driven DNAm for genetic association testing with complex traits. **a** In a reference sample, a elastic net penalized multiple regression model is built between SNPs in *cis* of a given CpG site, and DNAm signal (blue). The linear combination of the inferred weights *w* at selected genotypes (encircled in yellow) represents the genetically driven estimate of DNAm signal (gray). **b** The genetic model is used to estimate genetically driven DNAm in independent individuals, from observed genotypes; this estimate can be tested for association with a sample’s trait. In case genotypic data are not accessible (**c**), the association statistic can be approximated using the model’s weights, the trait GWAS summary statistics (SNP to trait association), and the covariance structure of model’s SNPs inferred from a reference sample (different blue dot sizes represent different covariance levels between pairs of SNPs in the reference sample). Figure 1 from Gusev et al.^7^ served as a template for this figure

We estimated, under an additive genetic model, the genetically driven proportion of DNAm at a given CpG site, as a linear combination of SNPs in *cis* of that site. Starting from a reference dataset of samples for which both methylation and genotypic data are measured, the weights of this linear combination can be obtained using a multiple regression approach between SNPs and corresponding DNAm. In analogy to the approach adopted previously for gene expression^4^, we opted for a elastic net penalized multiple regression method^27^ to infer SNP weights of the DNAm estimators (Fig. 1a). This method comes with the advantage of performing marker selection, thus providing sparse solutions. Subsequently, the genetically driven DNAm signal can be estimated in independent individuals as the linear combinations of the inferred weights and observed genotypes (Fig. 1b). In this independent sample, the derived genetic estimate of DNAm at a given CpG is amenable to genetic correlation testing with the phenotype under study (Fig. 1b).

Our reference dataset comprised *N* = 533 healthy young adults (BASEL1 dataset, see Materials and methods), who underwent both whole-blood methylomic profiling and genome-wide SNP assessment. Prior to analysis, the DNAm signal was adjusted for technical and biological confounders (see Materials and methods), and genotypes were imputed using the Michigan imputation server^26^ (https://imputationserver.sph.umich.edu/index.html, see Materials and methods).

In the reference sample, a elastic net model was trained between common *cis*-SNPs (MAF > 0.05, located within ±1 Mbp of a CpG site) and adjusted DNAm signal at each individual CpG site (see Materials and methods). From 395,014 CpG sites investigated, a total of 236,923 non-null models (i.e., at least one site selected by penalized regression) could be fitted, with cross-validation *r*^2^ accounting on average for 6.9% of variance of the DNAm signal.

**Fig. 2:**
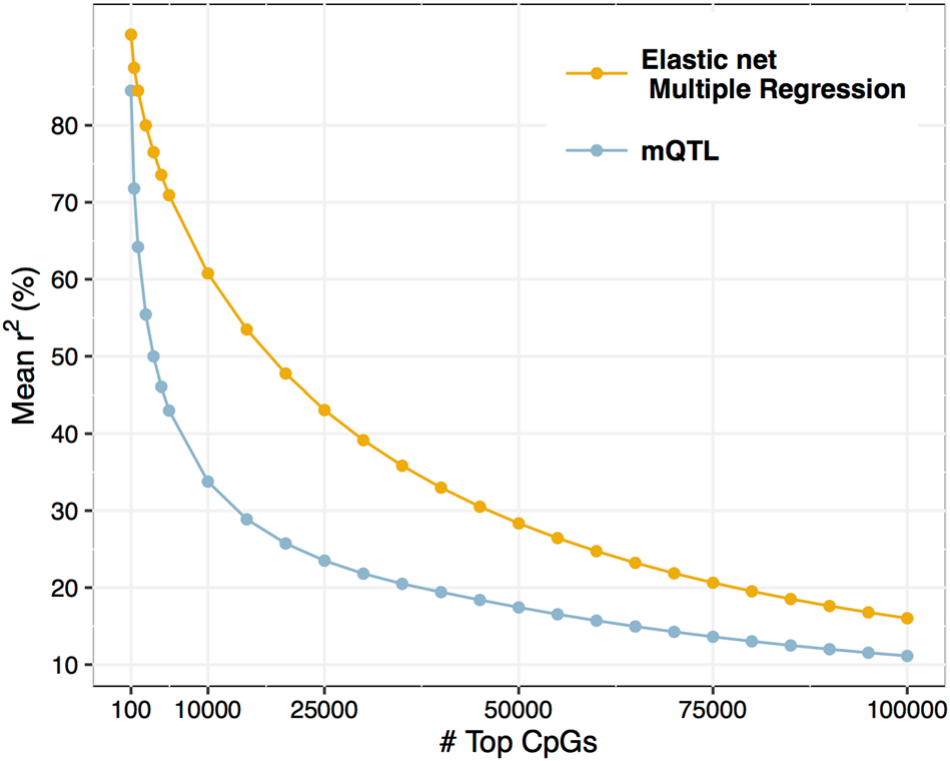
Comparison of average fraction of variance of DNAm variance explained by penalized multiple or univariate regression. Mean *r*^2^ corresponds to cross-validation performance of the elastic net model in the BASEL1 dataset, averaged across the top *n* CpGs (yellow), and *r*^2^ for the top identified mQTL per CpG averaged across the top *n* CpGs (blue)

Unlike univariate testing, the elastic net approach allows for simultaneous modeling of the joint effects of multiple *cis*-markers that are likely to impact on DNAm at a given CpG site^16^. We compared the fraction of variance of DNAm explained by the elastic net models (cross-validation *r*^2^) with the fraction of variance explained by the single best methylation quantitative trait loci (mQTL) identified at each CpG site. We observed substantial gain in average *r*^2^ retrieved by the multiple regression elastic net over single-marker univariate testing (Fig. 2). At the modeled CpGs (i.e., 236,923 non-null elastic net models), SNP-based heritability derived from recently published estimates in whole-blood samples^38^ averaged 9%. Thus, at these CpGs, the implemented performance of our models (6.9%) was close to the maximum variance in DNAm that can theoretically be explained by common SNPs. In addition, per-CpG cross-validation *r*^2^ showed high correlation with reported SNP-based (*r* = 0.53) and total heritability (*r* = 0.62) estimates across all modeled CpGs. Among CpGs for which no elastic net model could be fitted (*N* = 158,091 CpG sites), lower SNP-based heritability was observed, with an average of 4%.

**Fig. 3:**
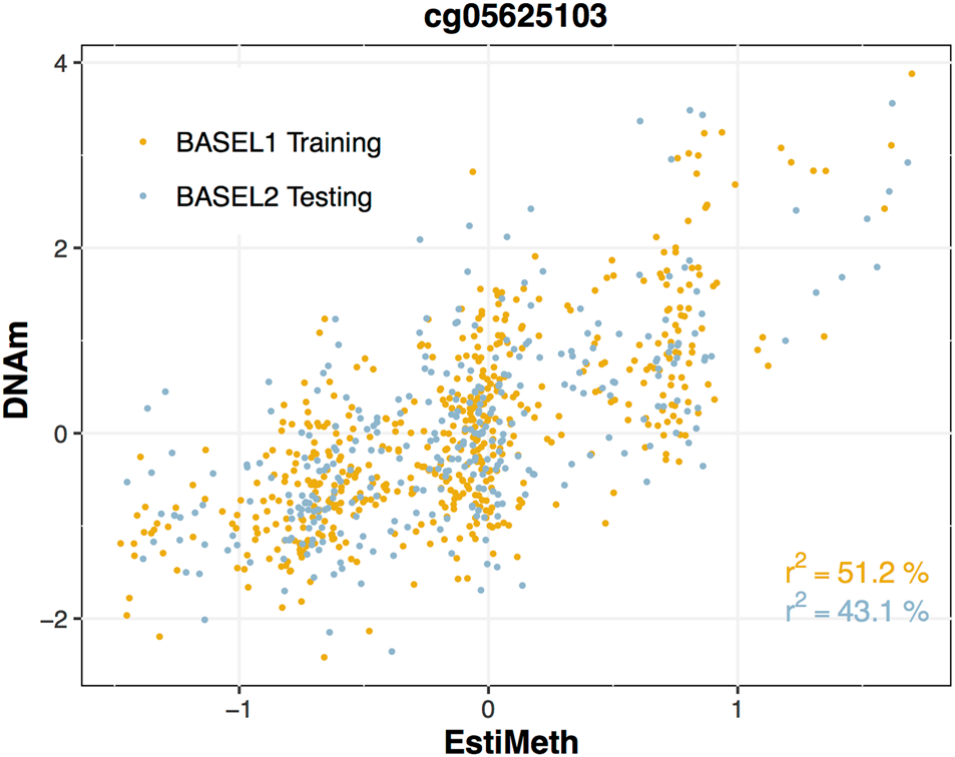
Example of a robust EstiMeth model. Horizontal axis represents the DNAm value estimated from the EstiMeth model at the CpG site. Vertical axis represents the observed DNAm value (adjusted for main confounders). The *r*^2^ is the fraction of variance of DNAm signal explained by the EstiMeth model (in %)

To assess the validity of the inferred genetic estimators we examined their accuracy to predict DNAm in an independent sample comprising whole-blood methylomic profiles from *N* = 319 healthy young adults (BASEL2 dataset, see Materials and methods). The correlation of model performance between training and testing samples across all modeled CpGs was high (*r* = 0.96) (Supplementary Figure 1). Moreover, the average performance (i.e., proportion of variance of the DNAm signal explained by the genetic models) of the testing sample (*r*^2^: 7.6%) was very close to the corresponding performance of the training sample (*r*^2^: 6.9%). These findings demonstrate high stability and generalization capability of the implemented genetic models. A set of 86,710 genetic models for DNAm estimation was identified as highly robust, showing significant (FDR < 0.05) and consistent correlation with DNAm across the two independent BASEL1 and BASEL2 datasets (see Materials and methods) (example shown in Fig. 3). These genetic estimators of DNAm are termed hereafter EstiMeth models.

The EstiMeth models were additionally tested in an independent dataset comprising whole-blood methylomic profiles from *N* = 288 elderly adults (ACD BONN dataset, see Materials and methods). We observed high correlation (*r* = 0.83) of EstiMeth model performance between the BASEL1 training and the ACD BONN dataset. In the ACD BONN dataset, the models accounted on average for 13.9% (mean testing *r*^2^) of DNAm (mean cross-validation *r*^2^: in the BASEL1 sample was 17.9% for EstiMeth models). This result further supports the robustness of the derived models in whole blood.

In real-life applications, not every SNP for a given EstiMeth might be available in the sample under study. On the other hand, EstiMeth SNPs in pair- or group-wise linkage disequilibrium might ensure robustness of the estimates also under incomplete SNP coverage. Therefore, we examined the performance of EstiMeth models after repeatedly discarding at random 10% of markers in the BASEL2 dataset (see Materials and methods). This resulted in an overall average distribution of *r*^2^ that was very close to the original distribution (Supplementary Figure 2), indicating high stability of most of the models under incomplete SNP coverage. We provide EstiMeth models together with summary statistics of their performance under varying missing rates, thereby enabling the estimation of the stability of each individual model.

We also examined whether inter-individual variability in whole-blood cell composition affects the EstiMeth models. In the two independent BASEL2 and ACD BONN testing datasets, we observed high consistency between the fraction of variance in DNAm accounted for by the models (*r* > 0.99) before and after adjustment for estimated cell type proportions (see Materials and methods) (Supplementary Figure 9), thus ruling out relevant confounding of the derived models by differences in whole-blood cell composition.

### Genetically driven DNAm is associated with gene expression of co-localizing genes

Each EstiMeth model corresponds to a CpG that is likely to be under strong genetic control. Given the role of DNAm in the regulation of gene expression^8^, we investigated the relationship between EstiMeth CpGs and expression levels of neighboring genes. Expression levels at ~20K genes were obtained for *N* = 408 individuals from the BASEL1 dataset (see Materials and methods).

First, we performed genome-wide association testing between DNAm and expression levels of genes located within ±1 Mbp of any CpG site (*N* = 397,731 sites). We identified 26,925 significant associations (FDR < 0.05), involving 6160 genes and 17,867 CpGs. Among these CpGs, we observed significant over-representation of EstiMeth CpGs (78% of EstiMeth CpGs among 17,867 CpGs associated with expression; 22% of EstiMeth CpGs across all investigated CpG sites; Fisher’s test *p* < 2.2e−16).

We also observed that EstiMeth CpGs, which were associated significantly with gene expression, were over-represented in shores (Supplementary Figure 3), which is in line with previous reports^39^. These results indicate that genetically driven (i.e., EstiMeth) CpGs are more likely to correlate with expression of co-localizing genes. This observation might also reflect the existence of shared genetic contributions between EstiMeth CpGs and the expression of their co-localizing genes.

**Fig. 4:**
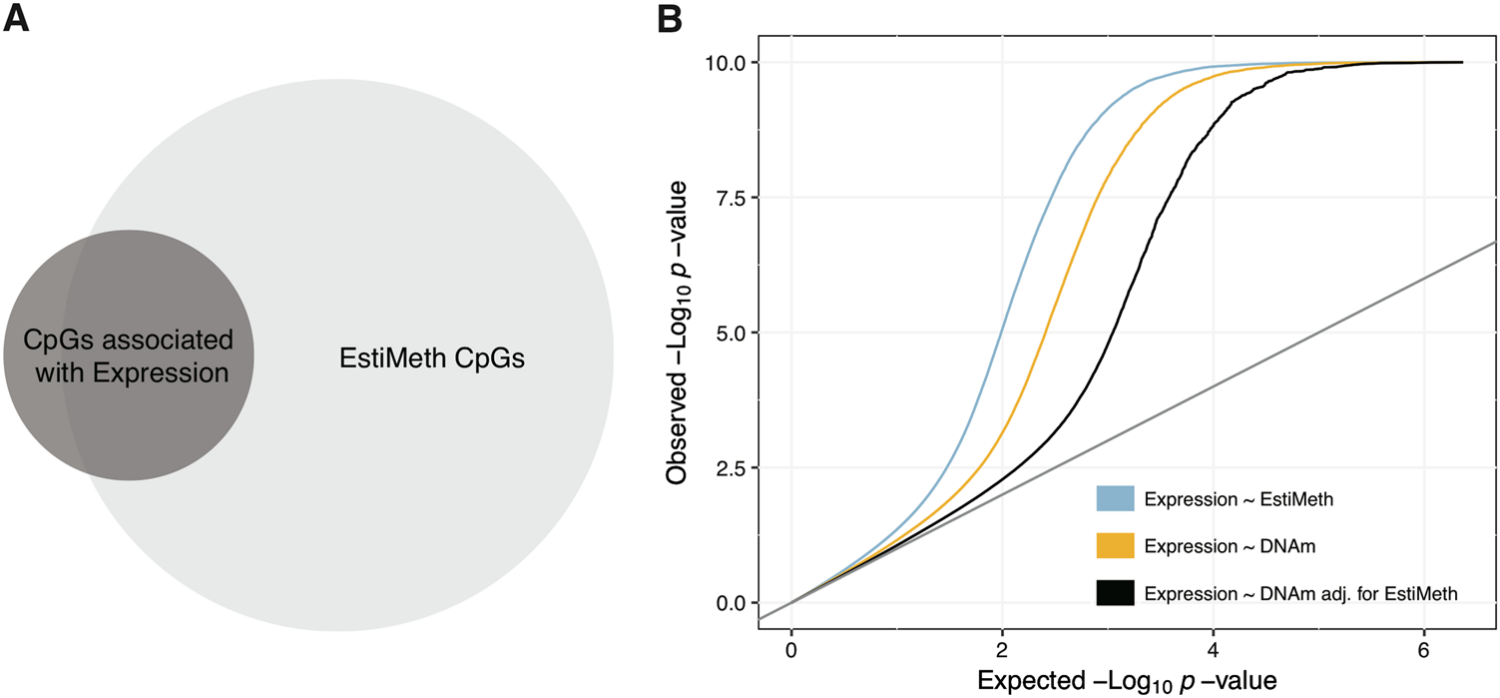
Relationship between DNAm, gene expression, and EstiMeth. **a** Overlap between CpGs associated with gene expression (FDR <0.05) and EstiMeth CpGs. **b** QQ plots for association between gene expression vs. EstiMeth estimates (blue), vs. DNAm (yellow), and vs. DNAm after adjustment for EstiMeth models (black). The *p*-values <1 × 10^−10^ are not shown

To test this hypothesis, we performed association testing between estimated DNAm values of each EstiMeth model and gene expression in *cis* (±1 Mbp). Given that gene expression was measured in the BASEL1 dataset, all EstiMeth models were re-implemented using the independent BASEL2 dataset as reference to prevent overfitting (see Materials and methods). Subsequently, a total of 2 million EstiMeth gene pairs were tested for association. We observed substantial deviation of genetic association signals from the null uniform distribution (Fig. 4b), with particular over-representation of large effect sizes. To further test whether EstiMeth models account for part of the shared variance observed between DNAm and expression, we also examined the DNAm expression associations after regressing out the effect of EstiMeth estimated DNAm values. We observed a consistent and substantial decrease of detected association signals (Fig. 4b). Notably, within CpG-gene pairs identified as genome-wide significant (FDR < 0.05), the average fraction of shared variance between DNAm and expression traits dropped from *r*^2^ = 9.6% to 2.3% after adjustment for EstiMeth model effects. These results support the existence of shared genetic contribution between DNAm and gene expression in *cis*, captured by the EstiMeth models.

Interestingly, the fraction of expression variance explained by the EstiMeth models was on average higher than the corresponding fraction explained by the DNAm signal alone (all EstiMeth CpG-gene pairs: *r*^2^ = 0.6% vs. *r*^2^ = 0.4%, Student's *t*-test *p*-value <2.2 × 10^−16^; genome-wide significant CpG-gene pairs: *r*^2^ = 16.9% vs. *r*^2^ = 9.6% Student's *t*-test *p*-value <2.2 × 10^−16^) (Fig. 4b). This suggests that the EstiMeth models are also likely to include SNPs having DNAm-independent effects on gene expression in *cis*. Of note, this increase in shared variance was mostly observed for CpGs located nearby the transcription start site of their associated gene (Supplementary Figure 4), a genomic location more likely to harbor *cis*-eQTLs^40^.

### Genetic correlation testing based on GWAS summary statistics

Provided availability of individual genotypic data in a given study sample, EstiMeth values can be readily obtained as the linear combination between the weights provided herein (derived from the BASEL1 dataset) and the observed SNPs (Fig. 1b).

Yet, genotypic data from large-scale genome-wide association studies are often not directly accessible. Recently, methods have been proposed that allow imputation of association statistics between genetic estimates of gene expression and a given trait, based solely on GWAS summary statistics^7,29^. Based on this body of work, we extended the EstiMeth models to a ‘MetaMeth’ approach that allows genetic correlation testing from GWAS summary statistics (Fig. 1c) (see Materials and methods). The *t*-value for association between the EstiMeth estimated DNAm values and the trait can be approximated using simultaneously: (1) EstiMeth SNPs’ weights, (2) the standardized GWAS summary statistics (i.e., results from SNP to phenotype association), and (3) the covariance structure of the SNPs included in the EstiMeth model (see Materials and methods) (Fig. 1c). The implementation relies on the covariance structure from a reference population, provided it is genetically congruent to the population under study (Fig. 1c). This latter assumption represents a critical issue of the MetaMeth implementation, as slight shifts between the reference and actual population structures may potentially induce biased estimates. In order to mimic such discrepancies, we systematically assessed the validity of the MetaMeth approach on the BASEL2 sample, while using the SNP covariance structure inferred from the genetically close, yet not identical, 1000G European (EU) population. Of note, all EstiMeth models were re-trained on the BASEL1 dataset, restricted to SNPs present in both 1000G and BASEL1 datasets, yielding a total of 86,518 retained models (see Materials and methods).

**Fig. 5:**
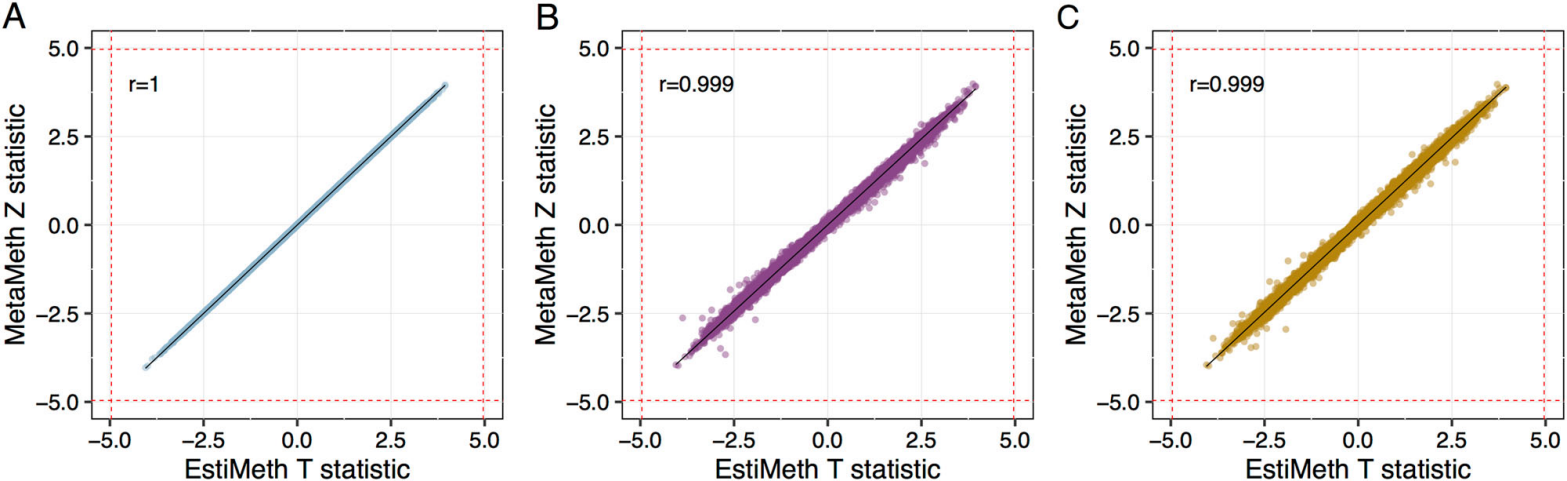
Comparison of EstiMeth and MetaMeth association statistics with height in the BASEL2 dataset. Each dot corresponds to an individual CpG included in EstiMeth models. Horizontal axis represents the *T-*statistic obtained from the correlation between sex-adjusted height and genotype-based EstiMeth estimate. The vertical axis represents the MetaMeth *Z*-statistic based on the SNP covariance structure observed within the BASEL2 dataset (**a**), from external independent BASEL1 dataset (**b**), and from external independent reference 1000G EU dataset (**c**). Red dashed lines represent critical statistics at Bonferroni adjusted significance threshold (*p* < 0.05/86,518)

Firstly, we examined the convergence of the EstiMeth (i.e., genotype-based) and MetaMeth (i.e., summary statistics-based) genetic correlation approaches by considering height as the complex trait under study. Under quasi-interchangeability of the two approaches, the MetaMeth *Z*-statistic should be close to the *T*-value obtained by testing directly association between height and the corresponding EstiMeth estimated DNAm values. Using the SNP covariance structure from the BASEL2 dataset, i.e., the actual population structure, the correlation between genetic correlation statistics was close to 1 (Fig. 5). We next used the SNP covariance structure derived from two independent population panels (i.e., BASEL1 dataset and 1000G EU dataset). The correlation between statistics obtained from the two approaches remained equal to 0.999 (Fig. 5), supporting the validity of the MetaMeth approach.

In a second stage, we estimated the Type I error rate of the MetaMeth approach. We performed a genome-wide MetaMeth scan on 1000 phenotypes generated from a normal distribution. The distribution of the minimum *p*-value obtained per run yielded a 5% quantile equal to 7.4e−07 (Supplementary Figure 5), which is above the Bonferroni-adjusted significance threshold for a given genome-wide scan (*p* = 5.8e−7). This indicates that under realistic settings the proposed MetaMeth yields conservative association statistics.

We next compared the power of the MetaMeth and EstiMeth methods. At each CpG we repeatedly generated a trait that was associated with EstiMeth estimated DNAm values at large effect sizes (i.e., *r*^2^ = 7.3% yielding an association detectable at 50% power under Bonferroni adjustment for multiple testing). Over all CpGs, the MetaMeth achieved lower average power (44.8%) as compared to the EstiMeth method (Supplementary Figure 6). Yet, we observed that for 16% of CpGs, the power of MetaMeth exceeded the power reached with the EstiMeth approach. These results indicate that in case of genuine association between EstiMeth and a trait, provided large effect sizes, the MetaMeth approach can lead to biased estimates of *T*-statistics, resulting in globally reduced power of detection.

### MetaMeth application to large-scale GWAS

We first applied MetaMeth on summary statistics obtained from the recently published schizophrenia PGC large-scale mega-analysis of GWAS results^32^. MetaMeth statistics were derived considering the 86,710 EstiMeth models implemented on the BASEL1 SNP panel and the corresponding BASEL1 covariance structure (see Materials and methods).

We observed a highly significant deviation of MetaMeth statistics from the null uniform distribution (Supplementary Figure 8). In particular, we identified a total of 469 associations withstanding genome-wide Bonferroni adjustment (unadjusted *p*-value < 5.78e−07). For the majority of these hits (*n* = 412, 87.8%), the corresponding EstiMeth model included at least one marker exhibiting a GWAS association *p*-value that would have reached a genome-wide GWAS Bonferroni adjustment significance threshold (*p* < 5e−08) (Supplementary Table 2). The majority of the identified CpGs (460 out of 469) lie within ±1 Mbp of 47 regions out of the 105 reported as independent autosomal genomic loci associated with schizophrenia (average genomic loci size: 202 kbp), or are found within the extended major histocompatibility complex (MHC) region (Supplementary Table 2).

We additionally applied MetaMeth on summary statistics results from well-powered GWAS, including AD^35^, RA^34^, IBD^33^ and Height^36^ (see Materials and methods). Significant associations (Bonferroni adjusted *p* < 0.05 for the number of CpGs investigated within a given MetaMeth scan) were identified in each GWAS investigated (AD: *n* = 31; RA: *n* = 402; IBD: *n* = 317; Height: *n* = 1934) (Supplementary Tables 3–6). The majority of identified signals arose from EstiMeth models including at least one genome-wide significant GWAS hit (between 92 and 95% across investigated GWAS). In addition, for almost all identified signals, at least one SNP reaching GWAS genome-wide significance (*p* < 5e−08) was located within ±1 Mbp of the CpG site (from 98 to 100% of significant hits across investigated GWAS, Supplementary Tables 3–6). A total of 90 CpGs identified as significantly associated with schizophrenia (19% relative to 469 CpGs) overlapped with CpGs associated with RA (*n* = 47) and/or Height (*n* = 46) with an important fraction located on chromosome 6 (66%) within or nearby the extended MHC locus (Supplementary Table 7). Overall, we observed a total of 154 CpGs identified in more than one MetaMeth scan, with an important representation of CpGs (55%) located within or nearby the extended MHC locus (Supplementary Table 7).

Thus, these results demonstrate that MetaMeth identified, among the large number of significant susceptibility variants for such polygenic disorders as schizophrenia, the ones that impact on disease risk probably through regulation of site-specific DNAm.

### Enrichment analysis for whole-blood and brain tissue-concordant CpGs

Recently, subsets of CpGs showing co-varying patterns between blood and brain tissue DNAm^41^ and enrichment of mQTLs among such CpGs^37^ were described. We thus investigated the distribution of co-variation patterns between whole-blood and brain tissue at EstiMeth CpGs, using recently published results derived from paired whole-blood Brodmann areas 7 (BA7), 10 (BA10) and 20 (BA20) samples across *N* = 16 subjects^37^. We observed significant enrichment of CpGs previously reported as concordant between whole-blood and brain tissues^37^ among EstiMeth CpGs (*n* = 18,910; 22% relative to 86,710 EstiMeth CpGs; permutation based *p* < 1e−04) (see Materials and methods). This suggests that a significant subset of the identified genetically driven CpGs show consistent DNAm pattern between blood and brain tissue.

## Discussion

We generated genetic estimators of epigenetic regulation—EstiMeth—that leverage genetic contributions to DNAm in whole blood to identify epigenetic underpinnings of complex traits. EstiMeth models together with MetaMeth and 1000G reference structure programs are made publicly available. By capitalizing on multiple co-localized genomic loci likely to impact on the DNAm signal, we identified a set of genetic estimators accounting on average for a modest, yet highly consistent fraction of variance in DNAm across independent samples.

Inter-individual variation in DNAm correlates with variation in expression levels of co-localizing genes possibly through shared genetic factors^11,12^. Here, integration of both methylomic profiles and gene expression data revealed that EstiMeth models accounted for a substantial fraction of the shared phenotypic variance between both molecular traits. This suggests that, in line with a recent report^42^, local genetic variations represent an essential factor underlying the observable inter-individual relationship between gene expression and DNAm at adjacent CpG sites. Importantly, the identified associations do not always imply direct causality between genetically driven DNAm and gene expression, as the EstiMeth models possibly include SNPs exerting independent effects on each trait^11,12^.

We also combined the genetic estimators for DNAm with recently proposed methods that allow applicability of these estimators to SNP summary statistics solely (i.e., in the absence of individual genotypic data)^7,29^. This approach, applied to recent large-scale GWAS results for schizophrenia^32^, resulted in the identification of 469 significant associations. This suggests the existence of shared genetic contributions between whole-blood DNAm and schizophrenia risk which is consistent with recent reports^43,44^. Of note, it cannot be excluded that the identified associations can also be partly driven by genetic loci exerting independent effects on each trait. A majority of the identified associations implicated genome-wide significant GWAS hits, whilst encompassing slightly less than half of the 105 genomic regions associated with schizophrenia^32^. In analogy to the findings obtained in the schizophrenia GWAS, the MetaMeth approach revealed significant associations with AD, RA, IBD, and Height, with the majority of the detected signals pointing to genome-wide significant GWAS markers. Importantly, each association suggests shared genetic contributions between a given trait and whole-blood DNAm variation at a specific CpG site. These results altogether highlight the potential of MetaMeth to decipher, from large-scale GWAS results, trait-associated loci that are putatively mediating their effect through methylation at given CpG sites, and to prioritize specific genomic loci for downstream functional validation.

Given the tissue-specific nature of DNAm, no direct and mechanistic link can be inferred between the identified MetaMeth association signals for schizophrenia and DNAm patterns observed in brain tissue. In line with previous results, we nevertheless observed that CpGs showing concordant DNAm patterns across whole-blood and brain tissue were enriched among genetically driven EstiMeth CpGs^37^. Thus, the provided genetic estimators might also serve as a valuable tool for the study of co-variation patterns between peripheral and brain tissue DNAm, whenever intra-individual multi-tissue samples are not available.

On the side of limitations, it should be stressed that the EstiMeth models were inferred and tested on moderately sized whole-blood samples. For about one-third of the investigated CpGs, characterized though by lower average SNP-based heritability, no elastic net model could be fitted from our training sample. Thus, additional local genetic contributions to DNAm might be detected with increasing sample sizes.

The fraction of variance explained by the EstiMeth models refers to DNAm signal after adjustment for main confounders. As for any omics dataset, such confounders are usually unknown and estimated empirically in a study-specific manner. This might ultimately impact on the fraction of variance in DNAm that can be retrieved by the derived genetic estimators. For instance, in our study, we observed that these estimators showed, on average, higher performance on a testing sample as compared to the training sample. Thus, although high stability was globally observed between performance of the models across the investigated samples, inference on multiple independent datasets, and multiple tissues, is warranted to fully appreciate their generalizability.

We also note that we generated a genetic estimator of DNAm at each single CpG site. Although this provides a straightforward way of annotating the models, it also results in certain redundancy of the estimators for highly correlated CpG sites. This in turn leads to a number of inferred estimators, which, unlike genetic estimators for gene expression, allow only for a moderate reduction of multiple testing burden in GWAS^4,7^. In addition, the derived genetic estimators were built on *cis* neighboring SNPs only. Although we globally observed high consistency of the inferred models’ performance with published SNP-based heritability estimates, the performance was on average lower than the reported common SNP heritability. This gap might be explained by additional trans genetic components likely to contribute to inter-individual variability in DNAm^15,16,44^. Improved accuracy might thus be achieved by extending the modeling to trans genetic components, as was recently shown for gene expression^45^.

We would also like to stress that the 450k array used in this study captures both methylation (5mC) and hydroxymethylation (5hmC). Even though the 5hmC mark remains strongly underrepresented in whole-blood tissue^46–49^, we cannot totally rule out a possible contribution of this mark to the detected signals reported herein.

Concerning the MetaMeth extension, we could derive empirical settings that showed appropriate control of Type I error in the investigated sample, yet at the cost of decreased average power of detecting genuine associations. Robustness of the derived statistic is also tightly linked to the genetic discrepancy between the reference and study population, which might not be easily evaluated in practice. This calls for assessment of the stability of the approach on larger independent samples from varying populations.

In conclusion, we provide genetic estimators for DNAm in whole blood that can effectively complement genetic estimators for gene expression to gain insight into the molecular underpinnings of complex traits.

## Electronic supplementary material

Supplementary Information

Supplementary Table 2

Supplementary Table 3

Supplementary Table 4

Supplementary Table 5

Supplementary Table 6

Supplementary Table 7
