# Supplementary material for: Genetic estimators of DNA methylation provide insights into the molecular basis of polygenic traits
Source: Transl Psychiatry. 2018 Jan 31;8:31. doi: 10.1038/s41398-017-0070-x (PMC5802460; doi:10.1038/s41398-017-0070-x)
Supplement: Supplementary file 1 — Supplementary Information [file 41398_2017_70_MOESM1_ESM.docx]

**SUPPLEMENTARY FIGURE 1: Elastic net models testing vs. training performance.** Horizontal axis denotes training cross-validation r^2^ performance in the BASEL1 dataset. Vertical axis represents performance of the models in the independent testing BASEL2 dataset. Dashed line represents regression line.

**
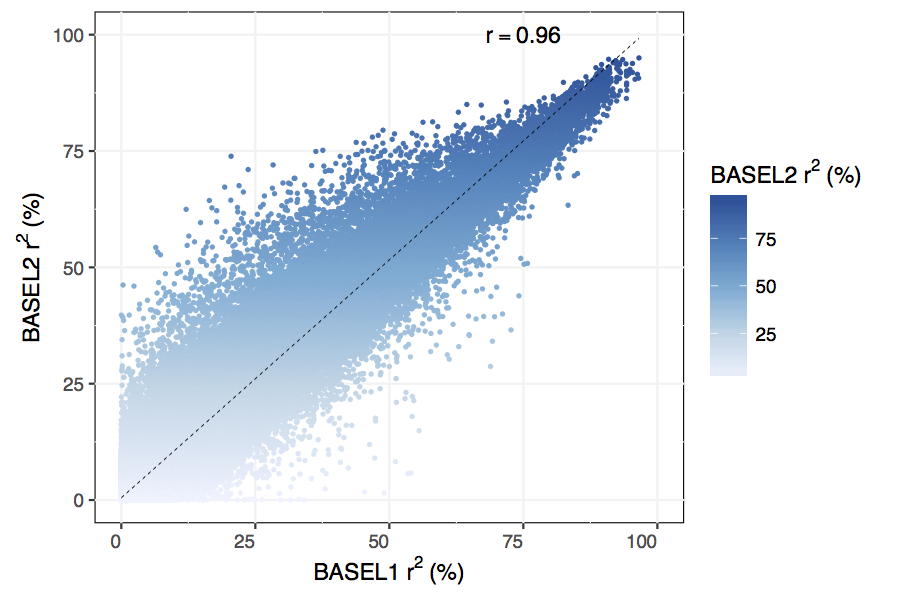
**

**SUPPLEMENTARY FIGURE 2: Distribution of EstiMeth models' testing r^2^ for varying genotyping missing rates.** EstiMeth models were evaluated on the BASEL2 testing sample with 10% (left panel) or 20% (right panel) of genotypes randomly discarded. For each model, the mean, and 5^th^ and 25^th^ quantiles of the r^2^ distribution obtained from 1000 runs was recorded. Graphs represent the density distribution of all EstiMeth models r^2^ (in %) for the complete models (red dashed line) or from simulations (blue, green and orange lines).

**SUPPLEMENTARY FIGURE 3: Distribution of EstiMeth CpGs associated with gene expression across genomic context**. Grey: Background CpGs (n = 397,947). Orange: EstiMeth CpGs associated with gene expression (n = 13,894). Blue: EstiMeth CpGs (n = 86,710). Green: CpGs associated with gene expression not included in EstiMeth CpGs (n = 3,973).

**SUPPLEMENTARY FIGURE 4:** **Comparison of shared variance between gene expression, DNAm and EstiMeth genetic contributions across genomic locations.** Horizontal axis: genomic location; bins correspond to the distance of a given CpG relative to its associated gene in kbp. TSS is defined as 1.5kbp upstream gene start. Vertical axis: fraction of shared variance (in %) between gene expression and EstiMeth (blue), DNAm (orange) or DNAm adjusted for EstiMeth effects (black). **A**: Average across ~2M EstiMeth CpG-gene association pairs. **B**: Average across EstiMeth CpG-gene association pairs identified as genome-wide significant (FDR<0.05).

**SUPPLEMENTARY FIGURE 5: Distribution of the minimum MetaMeth *p*-value per genome-wide scan under *H0****.* Phenotypes were drawn from a standard normal distribution (1000 runs). For each run, a MetaMeth analysis was performed across all modeled CpGs (n = 86,518) and the random phenotype in the BASEL2 dataset, using the covariance structures from 1000G EU population. The minimum *p*-value obtained across all CpGs was retained. The left panel represents the distribution of minimum *p*-values, obtained without penalization of the *Z* statistics (Equation 2, main text); right panel represents the distribution obtained using the penalty factor retained in the MetaMeth implementation.

**SUPPLEMENTARY FIGURE 6: Comparison of EstiMeth and MetaMeth power in the BASEL2 sample.** For each EstiMeth CpG, phenotypes were generated to be associated with EstiMeth estimate with 50% power of being detected (at α = *p* <0.05/86,518). The graph represents the density curves of power achieved across all CpGs, for EstiMeth (blue), MetaMeth using 1000G EU covariance structure (orange), and MetaMeth using actual sample's covariance structure (green).

**SUPPLEMENTARY FIGURE 7: Comparison of EstiMeth and MetaMeth association statistics for DNAm in the BASEL2 dataset.** Each dot corresponds to an individual CpG included in EstiMeth models. Horizontal axis represents the *T*-value obtained from the correlation between DNAm signal and EstiMeth estimate. In panel (**A**) the vertical axis represents *Z* statistic retrieved from Equation 1, based on the sample's SNPs covariance structure, which is equivalent to the *T*-value; in panel (**B**), the vertical axis represents the approximation MetaMeth Z statistic (Equation 2), based on the sample's covariance structure. In panels (**C**) and (**D**), the vertical axis represents the MetaMeth *Z* statistics based on SNPs covariance structure inferred from external BASEL1 and 1000G (EU) datasets respectively. Black line represents regression line.

**
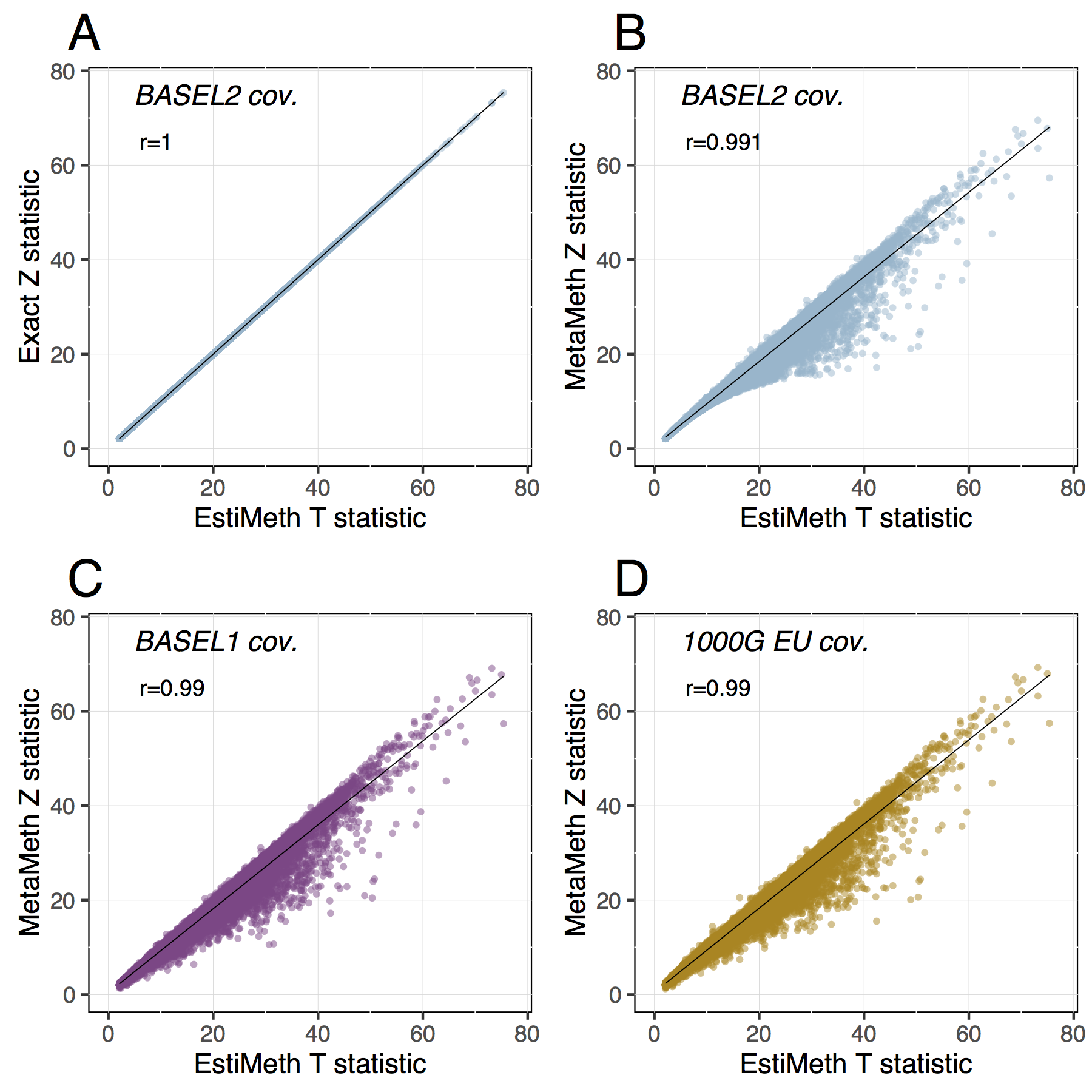
**

**SUPPLEMENTARY FIGURE 8: MetaMeth analysis of large-scale GWAS for schizophrenia**

**
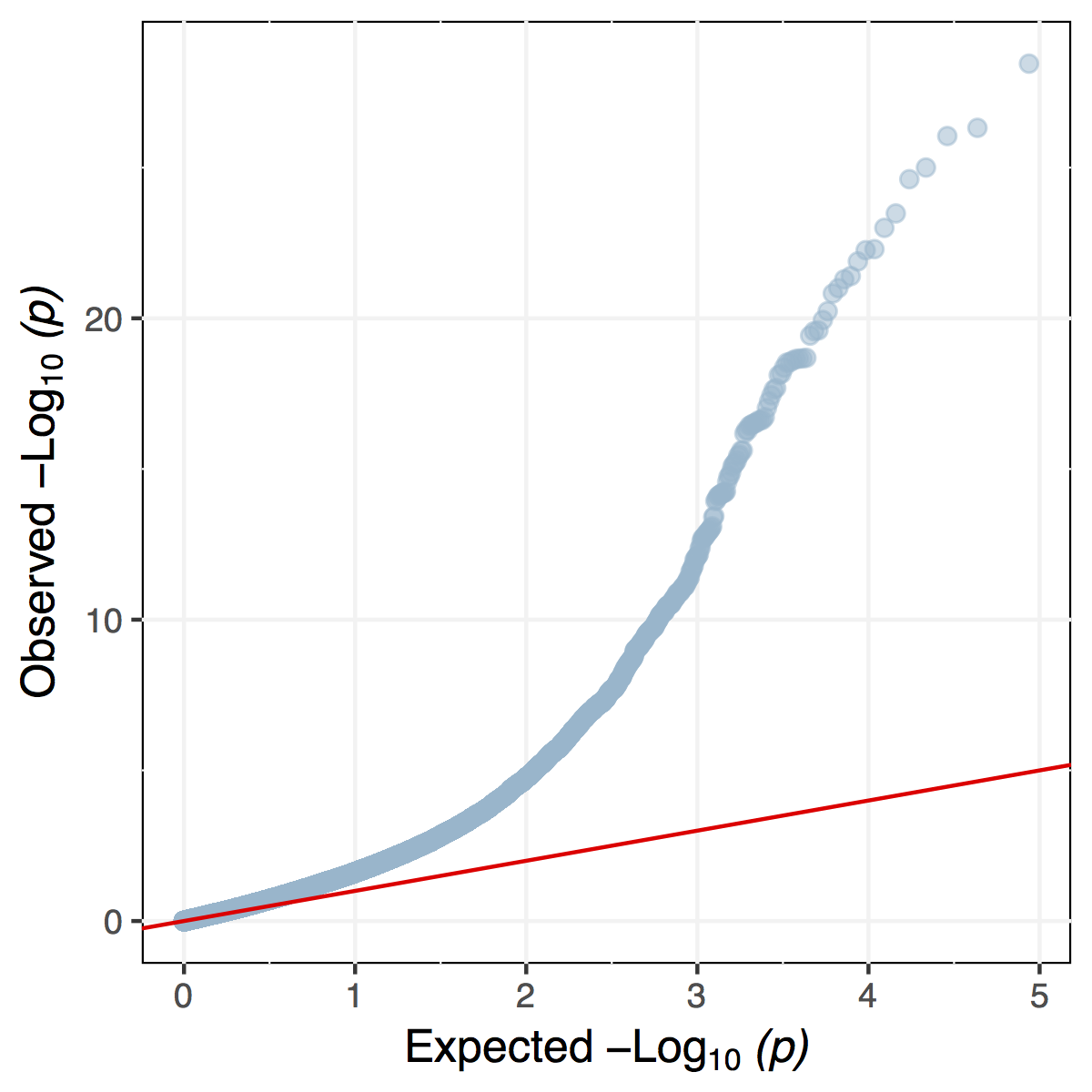
**

**SUPPLEMENTARY FIGURE 9:** Comparison of fraction of variance accounted by EstiMeth models before and after adjustment for cell types proportion

Horizontal axis denotes the fraction of variance accounted by the EstiMeth model (r^2^ in percent) on DNA methylation signal. Vertical axis denotes the fraction of variance accounted by the model after further adjustment for estimated cell type proportions. Left panel: BASEL2 dataset; Right panel: ACD BONN dataset.

**
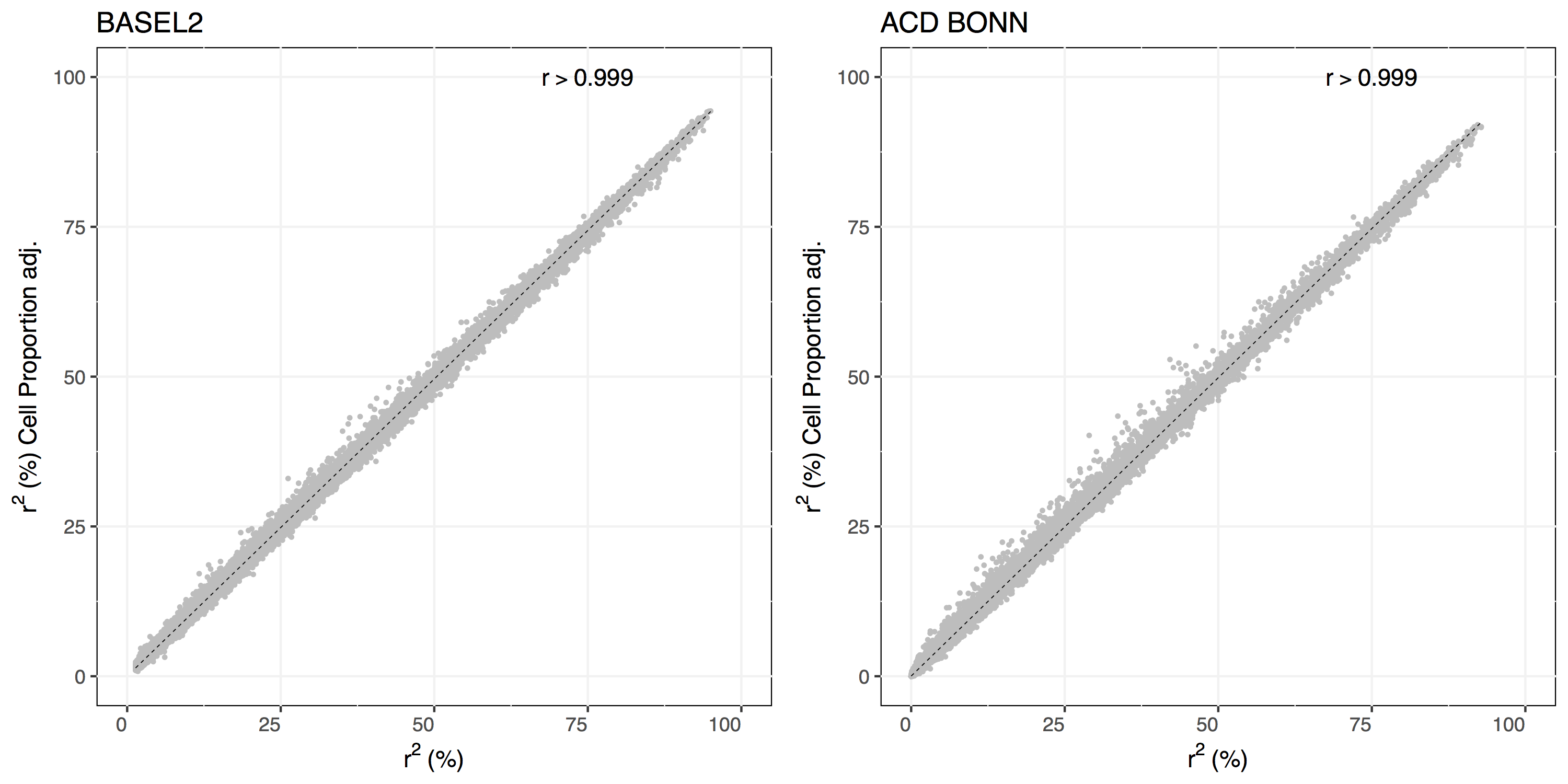
**

**SUPPLEMENTARY TABLE 1:** **Comparison of elastic net performance with/without standardization of the genotypes.**

|  | Genotypes  standardization | No Genotypes standardization |
| --- | --- | --- |
| Number of Non null models | 236,923 | 236,602 |
| r^2^ Training in % (M ± SD) | 6.9 ± 14.2 | 6.8 ±14.1 |
| r^2^ Testing in % (M ± SD) | 7.6 ± 15 | 7.4 ± 14.9 |
| Selected SNPs (M ± SD) | 25.6 ± 26.9 | 27.6 ± 30 |

All non-null models were considered. M: mean; SD: standard deviation.

**SUPPLEMENTARY TEXT:**

**Affymetrix HTA 2.0 array transcriptome analysis.** Total RNA was further isolated with the PAXgene Blood miRNA Kit (PreAnalytix, Switzerland). Following, a second, additional purification was performed with the miRNeasy Micro Kit (Qiagen, Germany). The concentration and quality of the RNA was determined using Nanodrop 2000 (ThermoScientific, USA) and RNA Nano 6000 Kit on Bioanalyzer 2100 instrument (Agilent, USA). Next, GLOBINclear™-Human Kit (Ambion, USA) was used for a non-enzymatic depletion of the alpha and beta globin mRNA starting from 1µg of total RNA preparations derived from whole blood, following a standard procedure. The concentration and quality of the “globin-free” RNA was assessed as described above. Following, the alpha and beta globin mRNA depletion was measured by qPCR. In brief: for reverse transcription, 350ng of total RNA was denaturized for 8 min at 70°C followed by ice incubation in the presence of 25ng Anchored Oligo(dT)20 Primer (Invitrogen, USA) and 75ng Random Decamers Primers (Ambion, USA). In the RT reaction, cDNA was generated in 25µl reaction using Super RT kit (HT Biotechnology, Santa Cruz, CA USA). Upon completion of the reaction, the volume was adjusted to 200µl in Lambda DNA solution (5ng/µl final concentration; Promega, Fitchburg, WI USA). The primers were designed against splice variants that contain alpha-Globin gene: alpha-Globin Forward: 5’- GCACGCGCACAAGCT-3’, and alpha-Globin Reverse: 5’- GGGTCACCAGCAGGCA-3’ (Microsynth, Switzerland). The expression levels were normalized to RPLPO gene (human large ribosomal protein) using the following primers: RPLP0-Ex3-4_FW, 5’-CTCTGGAGAAACTGCTGC-3’ and RPLP0-Ex3-4_RV, 5’-CTGATCTCAGTGAGGTCC-3’ (Sigma Aldrich, USA). qPCR was performed using the Power SYBR Green PCR Master Mix (Life Technologies, USA) according to standard recommendations, in 12µl final volume of reaction, using 2µl of cDNA template, on RotorGene 6000A instrument (Corbett Research Pty Ltd, Sydney Australia). Cycling conditions were as follows: 95°C, 60s – 40x (95°C, 3s - 56°C, 10s – 72°C, 4s) followed by a melting curve analysis (61°C to 95°C, rising by 0.7°C / 3s) to attest amplification specificity. Threshold cycles (crossing point) were determined using Rotor-Gene software version 6.1 (Corbett Research, Australia). RPLPO was selected as reference gene for normalization after we tested several candidate-reference genes, as had been previously described [1]. Expression levels were normalized using a geometric mean level of expression [1]. Fold differences were calculated using the delta-delta Ct method [2] with the help of qBasePlus software (Biogazelle, Ghent, Belgium).

Target synthesis was performed using Ambion® WT Expression Kit (Ambion, Life Technologies, USA) starting from 250ng of high-quality “globin-free” RNA, following the standard procedure. Next, 5.16µg of target cDNA was further labeled and prepared for hybridization with the GeneChip® WT Terminal Labeling and Hybridization Kit (Affymetrix, USA). The prepared samples were loaded on Affymetrix GeneChip Human Transcriptome Array 2.0 (Cat# 902162) and hybridized for 16 hours (45°C, 60rpm) in Hybridization oven 640 (Affymetrix, USA). The arrays were washed and stained on Fluidics Stations 450 (Affymetrix) by using the Hybridization Wash and Stain Kit (Affymetrix, USA) under FS450_0001 protocol. The GeneChips were processed with an Affymetrix GeneChip Scanner 3000 7G (Affymetrix, USA). DAT images and CEL files of the microarrays were generated using Affymetrix GeneChip Command Control software (Affymetrix, USA). In order to account for technical inter-array variation we performed a full quantile-normalization; feature quantification was conducted using a median-polish on transcript-level according to the HTA 2.0. lib-set-version 0.3. (Affymetrix Power Tools version: 1.16.0). Cross-platform validation of genotyping and expression data was assessed using the MixUpMaper algorithm [3].

**References**

1. Vandesompele J, De Preter K, Pattyn F, Poppe B, Van Roy N, De Paepe A, et al. Accurate normalization of real-time quantitative RT-PCR data by geometric averaging of multiple internal control genes. *Genome Biol.* 2002;**3**:RESEARCH0034.

2. Pfaffl MW A new mathematical model for relative quantification in real-time RT-PCR. *Nucleic Acids Res.* 2001;**29**:e45.

3. Westra H-J, Jansen R, Fehrmann R, te MeerMan G, van Heel D, Wijmenga C, et al. MixupMapper: correcting sample mix-ups in genome-wide datasets increases power to detect small genetic effects. *Bioinformatics* 2011;**27**:2104–11.
